# World Water Day — March 22, 2014

**Published:** 2014-03-21

World Water Day, sponsored by the United Nations, is observed each year on March 22. This year, World Water Day focuses on water and energy. Water and energy are closely linked. Worldwide, 1.3 billion persons currently live without electricity (1), 780 million lack access to safe drinking water, and 2.5 billion are without sanitation (2).

To some degree, water is crucial to produce, transport, and use all forms of energy, and these activities variously affect water resources. Demand for freshwater and energy will continue to increase significantly over the coming decades, presenting major challenges and straining resources in nearly all regions. This is especially true in developing and emerging economies. Better understanding of the linkages between the water and energy sectors can lead to improved coordination among policymakers, planners, and others in providing and obtaining more efficient and cost-effective water and energy services.

Additional information about World Water Day and ideas on how to get involved are available at http://www.unwater.org/worldwaterday. Information on CDC’s efforts to ensure global access to improved water, sanitation, and hygiene resources is available at http://www.cdc.gov/healthywater/global.
